# Supplementary material for: Association between oral microbiome and five types of respiratory infections: a two-sample Mendelian randomization study in east Asian population
Source: Front Microbiol. 2024 Apr 10;15:1392473. doi: 10.3389/fmicb.2024.1392473 (PMC11039966; doi:10.3389/fmicb.2024.1392473)
Supplement: Supplementary file 1 [file Data_Sheet_1.zip › Supplementary Materials/MR plots of saliva/Chronic sinusitis/s__Streptococcus_mitis_AT_mgs_1319/funnel.pdf]

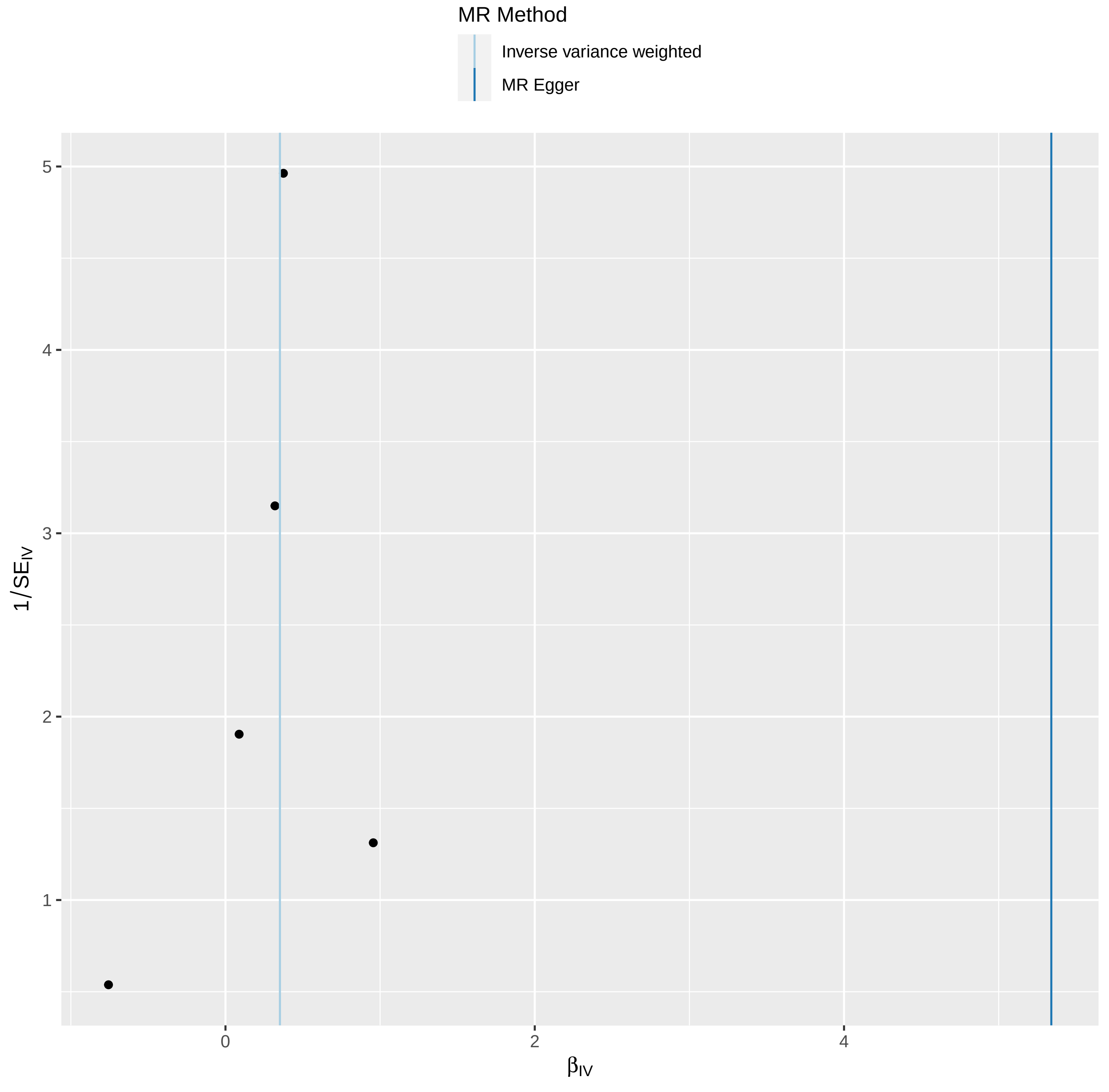
MR Method
Inverse variance weighted
MR Egger
5
4
3
2
1
0
2
4
$1/SE_{IV}$
β_{IV}
